# Draft Genome Sequence of *Streptococcus pyogenes* Strain M3KCL

**DOI:** 10.1128/genomeA.00610-17

**Published:** 2017-06-29

**Authors:** Conrrad Nicholls, Andrew Kump, Sarah Ford, Rusty Gonser, Kyu Hong Cho

**Affiliations:** a Department of Biology, Indiana State University, Terre Haute, Indiana, USA; b TCGA (The Center for Genomic Advocacy), Indiana State University, Terre Haute, Indiana, USA

## Abstract

We present here the draft genome sequence of *Streptococcus pyogenes* strain M3KCL. The assembly contains 1,864,059 bp in 60 contigs. This strain is an M3 strain close to MGAS315 but produces SpeB. It was isolated from the blood of a human patient with an invasive infection in 2009.

## GENOME ANNOUNCEMENT

*Streptococcus pyogenes*, also known as group A streptococcus (GAS), is a strict human pathogen and causes diverse diseases, from mild, self-limiting superficial infections to toxigenic or invasive diseases, which include strep throat, impetigo, scarlet fever, streptococcal toxic shock syndrome, rheumatic fever, and necrotizing fasciitis (1). Almost 10% of children carry this pathogen in their pharynx without signs or symptoms (2). We sequenced a serotype M3 strain isolated from the blood of a human patient. Often, serotype M3 strains are associated with an epidemic of invasive infections with a high mortality rate (3). The genome sequence of strain M3KCL is similar to that of strain MGAS315, a serotype M3 strain whose genome sequence is available publically. Unlike MGAS315, however, this strain secrets the cysteine protease SpeB, a major virulence determinant in the pathogenesis of GAS. It appears that *S. pyogenes* M3KCL has not developed antibiotic resistance yet. It is sensitive to most antibiotics, if not all, including ampicillin, clindamycin, cefazolin, erythromycin, cefoxitin, penicillin, cefotaxime, vancomycin, levofloxacin, tetracycline, ciprofloxacin, and teicoplanin.

The strain was grown in THY media overnight, and the chromosome was purified using a GenElute bacterial genomic DNA kit (Sigma Chemical Co., St. Louis, MO, USA). High purity of the chromosomal DNA was confirmed by running an agarose gel and measuring the ratio of OD_260_/OD_280_ (>1.8) using an Eppendorf BioSpectrometer. The purified chromosomal DNA was then sequenced using the Ion Torrent personal genome machine (PGM) according to the company’s specified protocols. The sequence information was available for export through the PGM database and obtained in the FASTQ format for annotation as a paired-end read library.

We utilized the Department of Energy’s Kbase system and the biological subunit application “Annotate Microbial Genome” for contig generation and gene annotation. First, the sequenced genome was taken from the PGM database and uploaded into Kbase in FASTQ format. The annotation and assembly application was run and the data were available for export in JSON and GenBank formats. The annotated and assembled product contained 60 total contigs (46 large contigs, after excluding those less than 500 bp), with a total length of 1,864,059 bp and a total G+C content of 38.42%. The largest contig available was 516,202 bp. The “spades_contigs” assembly pipeline method yielded the highest score out of the three assembly methods used within the Kbase system. The *S. pyogenes* strain HSC5 was used as both a sequencing and assembly reference, and the predicted genome size of M3KCL was similar to HSC5 at 1.8 Mb. Other *S. pyogenes* strains, such as MGAS315, SSI-1, and M1GAS, also shared a genome size similar to that we observed. The annotated shotgun sequence was exported as a GenBank file for deposit in NCBI’s WGS portal.

### Accession number(s).

The shotgun genome sequence reported here has been deposited at GenBank under accession number MTSW00000000.
